# Comparison of Efficacy of the Combination of Topical Tazarotene Gel and Oral Fluconazole Versus Oral Fluconazole Monotherapy in the Treatment of Onychomycosis

**DOI:** 10.7759/cureus.78265

**Published:** 2025-01-30

**Authors:** Hina Aslam, Sulaiman S Muhammad, Afshan Saeed, Muhammad A, Rami Ghafar, Suhib Abushehab, Sameen S Sawdagar, Yousra Abdulkhaliq, Aws K Mohammad Rababah, Gurnam Virdi

**Affiliations:** 1 Pharmacology, King Edward Medical University, Lahore, PAK; 2 General Practice, University Hospitals Birmingham, Birmingham, GBR; 3 Dermatology, Pak Emirates Military Hospital, Rawalpindi, PAK; 4 Medicine and Surgery, Pak Emirates Military Hospital, Rawalpindi, PAK; 5 Internal Medicine, University Hospitals Bristol and Weston NHS Foundation Trust, Bristol, GBR; 6 General Internal Medicine/General Surgery, Southeast University, Nanjing, CHN; 7 Dermatology, Benazir Bhutto Shaheed Hospital, Quetta, PAK; 8 Internal Medicine, Coventry University, Covnetry, GBR; 9 Dermatology, Academy of Aesthetic Medicine, Sheffield, GBR

**Keywords:** antifungal efficacy, antifungal treatment, onychomycosis, oral fluconazole, tazarotene

## Abstract

Background: Onychomycosis is a chronic fungal infection of the nails of toes and fingers, with an ongoing search for appropriate and more officious treatment modalities because the currently applied regimens are far from perfect.

Objective: The objective of this study was to compare the efficacy of the combination of topical tazarotene 0.1% gel and oral fluconazole with weekly oral fluconazole monotherapy in the treatment of OM.

Methodology: This quasi-experimental study was conducted over three months in the outpatient dermatology clinic of a tertiary healthcare facility in Pakistan. A total of 60 patients fulfilling the inclusion criteria were included. A detailed history, dermatological examination, and Onychomycosis Severity Index (OSI) were implemented in each patient to record the severity of their disease and treatment outcome. The patients were randomized into two groups i.e. Group A, where a weekly oral fluconazole tablet of 150mg and daily topical tazarotene 0.1% gel were administered, and Group B in which a weekly oral fluconazole tablet of 150mg alone was administered for three months each. The potassium hydroxide mounts from nail materials were performed at the end of the treatment period in all the patients. Data analysis was carried out using IBM SPSS Statistics for Windows, Version 23 (Released 2015; IBM Corp., Armonk, New York, United States).

Results: Of the total, 36 (60%) were male with a mean age of 46.47±10.60 years and 24 (40%) were female with a mean age of 46.08±10.16 years. Following the comparison between the two groups, the combination therapy had higher efficacy and better treatment success (70%, n=23) compared to fluconazole monotherapy (56.6%, n=17), in terms of the fall in the degree of OSI, clinical evaluation, and KOH preparation after treatment (p-value <0.001, 0.001, and 0.03, respectively).

Conclusions: The efficacy of oral weekly fluconazole combined with daily topical tazarotene 0.1% gel is significantly higher than that of weekly fluconazole monotherapy in the treatment of OM.

## Introduction

Onychomycosis (OM) is a well-documented cutaneous ailment where the infection of the nail unit by various types of fungi namely dermatophytes, non-dermatophyte molds, or yeasts is noted. Being one of the most common causes of nail infections, the important clinical features of OM include discoloration (yellow or brown) and nail plate thickening [1,2]. Other features include green, violaceous, or black discoloration, subungual hyperkeratosis, and dermatophytoma formation [3,4]. It can affect any part of the nail unit.

Dermatophytes are the main cause of toe and fingernail OM with *Trichophyton mentagrophytes and Trichophyton rubrum, Epidermophyton floccosum, Trichophyton tonsurans, Trichophyton verrucosum (T. verrucosum), *and* Microsporum species (Microsporum Sp.*) [2-4] being the most common causes.

The notable non-dermatophyte molds inflicting OM include *Aspergillus species, Scopulariopsis sp., Fusarium sp., Neoscytalidium sp., Syncephalastrum sp., and Acremonium sp. *[5-7]. An uncommon cause of OM includes yeasts (majority by *Candida albicans*), especially in immune-compromised patients [7]. Despite incurring a considerable level of morbidity in the affected patients [1,2], the current strategies to treat OM are far from perfect.

Currently, a host of patient and environment-related factors are causing a rise in the prevalence of OM. Some of these factors include modern occlusive footwear, a longer life expectancy, increased urbanization, increased prevalence of obesity, etc. [1,8,9]. Oral antifungal drugs (terbinafine, itraconazole, and fluconazole) are considered the mainstay of treatment; however, this modality isn’t devoid of its considerable share of demerits, which include, but not limited to, a high incidence of anorexia, taste disturbance, headaches, diarrhea, vomiting, dermatitis, deranged liver and renal function tests, etc. [10,11].

A higher treatment failure rate, frequent recurrences, poor compliance due to prolonged treatment duration, and increased cost are the other important hurdles in the management of OM [1,8,9]. So, an ongoing search for optimal treatment modalities that are devoid of such demerits or at least have minimal shortfalls is only a natural pathway for current researchers.

Oral fluconazole has been used for the treatment of OM in different countries in various age groups [8-11]. As a monotherapy, oral fluconazole is reported to have similar problems as described above. Various topical agents such as terbinafine, amorolfine, and efinaconazole. have been utilized with variable results [12-14]. When used as monotherapies, these topical agents are far less effective than their oral counterparts, largely due to their inadequate penetration of the nail plate [12-14]. A rational approach is to combine oral antifungals with topical agents, which may increase the frequency of treatment success [12].

The role of topical tazarotene, a retinoid from the acetylenic class, has been explored by a few studies in the treatment of OM. It has an antifungal action through its anti-inflammatory and immunomodulatory effects as well as by decreasing the rate of hyper-keratinization through a fall in the hyper-proliferation associated with keratin 16 & 06 [14].

Although a few studies have directly compared the efficacy of topical tazarotene 0.1 % gel with weekly oral fluconazole, there is a relative dearth of studies that compare the combination of topical tazarotene 0.1 % gel and weekly oral fluconazole with fluconazole monotherapy in the treatment of OM.

The aim of this study was to compare the efficacy of the combination of topical tazarotene 0.1 % gel and weekly oral 150mg fluconazole with weekly fluconazole monotherapy in the treatment of OM in a Pakistani cohort.

## Materials and methods

This quasi-experimental study was conducted in the outdoor department of dermatology of a tertiary healthcare hospital (Pak Emirates Military Hospital, Rawalpindi) from January 2023 to June 2023, following due approval from the institutional ethical committee. Following explicit informed written consent from all patients, 60 patients fulfilling the inclusion criteria from the Department of Dermatology were included in the research. Male and female patients of age 15-65 years, suffering from OM with a duration of > 3 months, who did not receive any topical or systemic anti-fungal therapy in the preceding two weeks and three months, respectively, with a positive potassium hydroxide mount of the nail clippings/scrappings, and willing to attend follow-up appointments, were included in the study. Patients with dermatological ailments that mimicked or presented with OM such as psoriasis, lichen planus, and nail dystrophy of various local and systemic causes other than OM were excluded from the study. Individuals with a history of active systemic ailments like ischemic heart disease, diabetes, endocrinopathies, hypertension, immunosuppression, positive pregnancy on laboratory tests, and those unable to maintain a follow-up were also excluded from the study.

A detailed history of all the volunteers and their general as well as dermatological examination were carried out. The Onychomycosis Severity Index (OSI) was employed to assess the severity of the condition in each patient [15] and was calculated at the end of the treatment period for both groups. The OSI is obtained by scoring the area of involvement (range, 0-5) and multiplying it with the score for the proximity of OM to the nail matrix (range, 1-5). In case of the presence of greater than 2 mm of subungual hyperkeratosis or a longitudinal streak/patch (i.e. dermatophytoma), ten points are added. The OSI was recorded as mild OM (1-5), moderate (6-15), and severe (16-35). At each visit, potassium hydroxide stains were also conducted on the nail scraping or clippings. The KOH mount preparation, clinical evaluation of OM, and calculation of the OSI were done by a trained team of dermatologists whose methods of assessment were standardized via a collective workshop before the commencement of the study, to make the assessment process more consistent. 

Randomization was conducted through sequentially numbered opaque envelopes generated from a random numbers table into two groups (Group A & Group B) of 30 patients each.

Each patient was assigned a number at enrolment which defined a study drug assignment (Topical Tazarotene 0.1% gel twice daily & weekly 150mg tablet Oral Fluconazole vs weekly 150mg tablet Oral Fluconazole Monotherapy). In Group A, 30 patients received twice daily topical Tazarotene 0.1% gel & weekly 150mg Oral Fluconazole for three months whereas in Group B, 30 patients received weekly Oral 150mg Fluconazole only for the same duration. Each patient was followed up for another three months on a monthly basis to evaluate for any evidence of nail discoloration, dystrophy, onycholysis, subungual hyperkeratosis, the resolution of the presenting symptoms and signs of OM, and a fall in the OSI by trained researchers.

Data was analyzed by using IBM SPSS Statistics for Windows, Version 23 (Released 2015; IBM Corp., Armonk, New York, United States). Baseline variables were analyzed descriptively using frequencies and percentages for qualitative variables and the mean with SD was calculated for quantitative variables. The chi-square test was used to compare the efficacy of both drugs with a p-value of ≤0.05 taken as significant.

## Results

Our study comprised a total of 60 individuals. Of the total, 36 (60%) were male with a mean age of 46.47±10.60 years and 24 (40%) were female with a mean age of 46.08±10.16 years.

The comparison between the two groups treated with either Topical Tazarotene 0.1% gel & weekly 150mg tablet Oral Fluconazole (Group A) or weekly 150mg tablet Oral Fluconazole Monotherapy (Group B) focusing on the degree of OSI and clinical response after treatment is shown in Table 1.

**Table 1:** Comparison of topical tazarotene and oral fluconazole using the onychomycosis severity index (OSI) and clinical response.

		Drug Groups		Total	p-value
		Topical Tazarotene & Oral Fluconazole Combined (Group A) n=30	Oral Fluconazole Monotherapy (Group B) n=30		
Post Treatment OSI (Performed in Each Patient at the End of the Treatment Period)	Cured	23 (70%)	17 (56.6%)	40 (66.6%)	<0.001
	Mild	4 (13.3%)	2 (6.6%)	6 (10%)	
	Moderate	1 (3.3%)	6 (20%)	7 (8.4%)	
	Severe	2 (6.6%)	5 (16.6%)	7 (10%)	
Total		30 (50%)	30 (50%)	60 (100%)	
Clinical Evaluation at the End of the Therapy	Complete Cure	23 (70%)	17 (56.6%)	40 (66.6%)	0.001
	Mild Response	5 (16.6%)	8 (26.6%)	13 (21.6%)	
	No Response	2 (6.6%)	5 (16.6%)	7 (11.6%)	
Total		30 (50%)	30 (50%)	60 (100%)	

A statistically significant difference indicated by the p-value (0.001) implied that the combination therapy involving topical tazarotene gel 0.1% with weekly 150mg oral fluconazole was far more efficacious than weekly oral 150mg fluconazole monotherapy when compared in terms of the level of OSI and clinical evaluation post-treatment. In the tazarotene-fluconazole arm, 70% (n=23) patients were declared healed compared to fluconazole monotherapy where around 57% (n=17) patients were declared healed on the basis of clinical examination at the end of the treatment period. A comparison of outcomes in both the arms evaluated via KOH testing is depicted in Table 2.

**Table 2:** Comparison of topical tazarotene and oral fluconazole combined vs fluconazole monotherapy regarding potassium hydroxide mount preparation after treatment.

		Drug Groups		Total	p-value
		Topical Tazarotene & Oral Fluconazole Combined (Group A) n=30	Oral Fluconazole (Group B) n=30		
KOH after Treatment	Negative	23 (76.6%)	19 (63.3%)	42 (70%)	0.003
	Positive	7 (23.3%)	11 (36.6%)	18 (30%)	
Total		30 (50%)	30 (50%)	60 (100%)	

The p-value (0.003) indicated that combination therapy involving topical tazarotene gel 0.1% with weekly 150mg oral fluconazole was more effective than weekly oral 150mg fluconazole monotherapy in obtaining a negative KOH mount.

## Discussion

OM is a chronic nail infection caused by dermatophytes, non-dermatophytes, and yeasts of various types. Good patient compliance is imperative for treatment success as the treatment of OM remains a prolonged and arduous one for the patients and clinicians due to its host of potential side effects [1,2,8].

In our study, the majority of the patients presented with a distolateral subungual-type OM followed by total dystrophic-type OM. Our study found that the combination therapy involving topical tazarotene gel 0.1% with weekly 150mg oral fluconazole was far more efficacious than weekly oral 150mg fluconazole monotherapy. A very limited amount of work has been done regarding this important comparison [13].

Earlier studies have reported a shred of conflicting evidence regarding the efficacy of topical tazarotene 0.1% gel as a monotherapy, in OM especially when compared to oral fluconazole. A study by Campione et al. [14] compared topical tazarotene 0.1% gel with tazarotene nail paint and reported an efficacy of 40% in those treated with the gel over a course of four weeks. On a similar note, a study by El-Salam et al. [13] reported a cure rate of 25% and a mild clinical response in 30% of the patients in the tazarotene-only arm. This study also described a favorable treatment outcome when tazarotene gel was combined with topical tioconazol (50%), as compared to tazarotene monotherapy (30%). This study thus indirectly corroborates our discourse that both tazarotene gel and oral antifungal medications may fare well when they are used in combination with each other than in monotherapy. With the logical assumption that the combination approach may also potentially allay the dilemma of antifungal resistance that dermatologists worldwide are currently grappling with, our study asserts the potentially positive outcomes of using a combination regimen compared to a monotherapy approach.

Oral fluconazole is being employed across Europe and America for the management of OM by clinicians, especially when intolerability of medications like itraconazole and terbinafine may be a concern [2,11].

In countries like Pakistan, weekly oral fluconazole is frequently employed for OM treatment, because of its easy dosage regimen and relative cost-effectiveness, although local studies exploring its efficacy as a single-dose regimen are sparsely conducted. The comparative data on the efficacy of fluconazole and tazarotene 0.1% gel combination regimen is even sparser. To the best of our knowledge, no clinical studies have been conducted in Pakistan to date on this comparison. The use of oral antifungal medications for the treatment of OM particularly when ≥ 50% of the nail is involved makes fluconazole an appropriate choice [1,9,11].

With previous studies reporting a better treatment outcome with oral fluconazole than with topical tazarotene 0.1% gel, the rationale of combining oral antifungals with topical agents, to increase the treatment success ratio becomes more and more plausible [8,12]. In a practical clinical environment where treatment of OM with monotherapy is faced with treatment failures, suboptimal response and even antifungal resistance, the addition of daily tazarotene gel to weekly oral antifungal therapy may serve as a useful adjunct option, potentially resulting in higher clinical cure rates.

Our study albeit, with a small sample size, highlights the need for further large-scale research where oral antifungal may be combined with topical tazarotene and possibly other topical agents with an antifungal role, for improving the clinical and mycological cure rates of OM. Based on our findings, we recommend the use of the combination of daily topical tazarotene gel 0.1% with weekly 150mg oral fluconazole for rapid and more efficacious treatment of OM. It is also pertinent to mention that since the nail plate is thinner and its growth rate is found to be faster in children than in adults, pediatric patients are expected to respond more rapidly to topical tazarotene 0.1% therapy compared to their adult counterparts; however, the safety and tolerability need to be ascertained first via pilot and subsequent large scale studies in the pediatric population where the tolerability and compliance of oral treatment modalities remain a big challenge [2,8,11].

The limitations of our study include a small sample size and our inability to perform pre- and post-treatment dermoscopy and fungal cultures on the affected nails to assess treatment outcomes in a better way. We tried to overcome these limitations by performing meticulous clinical as well as KOH mount examinations. Another limitation included our study’s inability to record the side effects of both treatment modalities. As all of the patients in the fluconazole arm were healthy individuals with no comorbidities, we didn’t expect any major adverse effects over the treatment period, due to the relatively high tolerability of fluconazole [11] although we did have a verbal inquiry regarding any complaints during the testament period. Another important limitation was that the pretreatment OSI was not calculated and compared in both arms and we monitored only the post-treatment OSI. Thus, we were not able to comment on whether the combination approach or monotherapy had different therapeutic outcomes when the levels of severity of OM were compared. Hence, we recommend further large-scale randomized clinical studies to record the dermoscopy, fungal culture examination, and the potential side effects of both treatment modalities for a better idea regarding their safety and efficacy especially in combination form.

## Conclusions

The combination of weekly 150mg oral fluconazole and daily topical tazarotene 0.1% gel application in the treatment of OM of toes and fingers may result in a rapid clinical response and higher treatment success rates than with weekly oral fluconazole alone. Clinicians should consider using this combination strategy in the treatment of OM.

## References

[1] Vlahovic TC (2016). Onychomycosis: evaluation, treatment options, managing recurrence, and patient outcomes. Clin Podiatr Med Surg.

[2] Lipner SR, Scher RK (2019). Onychomycosis: clinical overview and diagnosis. J Am Acad Dermatol.

[3] Joyce A, Gupta AK, Koenig L, Wolcott R, Carviel J (2019). Fungal diversity and onychomycosis: an analysis of 8,816 toenail samples using quantitative PCR and next-generation sequencing. J Am Podiatr Med Assoc.

[4] Ahmed R, Khoso BK, Uddin F, Rehman N, Sohail M, Hafiz S (2021). A cross sectional study to observe the diversity of fungal species in onychomycosis isolated from a tertiary care hospital in Karachi. J Pak Med Assoc.

[5] Pang SM, Pang JY, Fook-Chong S, Tan AL (2018). Tinea unguium onychomycosis caused by dermatophytes: a ten-year (2005-2014) retrospective study in a tertiary hospital in Singapore. Singapore Med J.

[6] Bombace F, Iovene MR, Galdiero M (2016). Non-dermatophytic onychomycosis diagnostic criteria: an unresolved question. Mycoses.

[7] Subramanya SH, Subedi S, Metok Y, Kumar A, Prakash PY, Nayak N (2019). Distal and lateral subungual onychomycosis of the finger nail in a neonate: a rare case. BMC Pediatr.

[8] Christenson JK, Peterson GM, Naunton M, Bushell M, Kosari S, Baby KE, Thomas J (2018). Challenges and opportunities in the management of onychomycosis. J Fungi (Basel).

[9] Gupta AK, Cernea M, Foley KA (2016). Improving cure rates in onycho mycosis. J Cutan Med Surg.

[10] Houšť J, Spížek J, Havlíček V (2020). Antifungal drugs. Metabolites.

[11] Gupta AK, Haas-Neill S, Talukder M (2023). The safety of oral antifungals for the treatment of onychomycosis. Expert Opin Drug Saf.

[12] Kably B, Launay M, Derobertmasure A, Lefeuvre S, Dannaoui E, Billaud EM (20221). Antifungal drugs TDM: trends and update. Therapeutic Drug Monitoring.

[13] El-Salam SS, Omar GA, Mahmoud MT, Said M (2020). Comparative study between the effect of topical tazarotene 0.1 gel alone vs its combination with tioconazole nail paint in treatment of onychomycosis. Dermatol Ther.

[14] Campione E, Paternò EJ, Costanza G (2015). Tazarotene as alternative topical treatment for onychomycosis. Drug Des Devel Ther.

[15] Carney C, Tosti A, Daniel R, Scher R, Rich P, DeCoster J, Elewski B (2011). A new classification system for grading the severity of onychomycosis: Onychomycosis Severity Index. Arch Dermatol.

